# Dynamic Ultrasound Assessment of the Anterior Tibial Translation for Anterior Cruciate Ligament Tears Diagnostic

**DOI:** 10.3390/jcm11082152

**Published:** 2022-04-12

**Authors:** Anca Gabriela Stoianov, Jenel Marian Pătrașcu, Bogdan Gheorghe Hogea, Bogdan Andor, Liviu Coriolan Mișcă, Sorin Florescu, Roxana Ramona Onofrei, Jenel Marian Pătrașcu

**Affiliations:** 1 Department of Orthopedics and Trauma, “Victor Babes” University of Medicine and Pharmacy Timisoara, 300041 Timisoara, Romania; anca.stoianov@umft.ro (A.G.S.); patrascujenel@yahoo.com (J.M.P.); hogeabg@yahoo.com (B.G.H.); andormed@yahoo.com (B.A.); miscal.liviu@yahoo.com (L.C.M.); florescusorin@yahoo.com (S.F.); 2 Department of Rehabilitation, Physical Medicine and Rheumatology, Research Center for Assessment of Human Motion, Functionality and Disability, “Victor Babes” University of Medicine and Pharmacy Timisoara, 300041 Timisoara, Romania

**Keywords:** dynamic ultrasound, ACL tears, anterior tibial translation, test–retest reliability

## Abstract

The aim of our study was to investigate the accuracy of dynamic ultrasound assessment of the anterior tibial translation, in diagnosing anterior cruciate ligament tears, and to assess its test–retest reliability. Twenty-three patients (32 ± 8.42 years; 69.56% males) with a history of knee trauma and knee instability participated in the study. Knee ultrasound was performed by an experienced orthopedic surgeon. The anterior tibial translation was measured in both knees and differences between the injured and uninjured knee were calculated. Side-to-side differences > 1 mm were considered a positive diagnosis of an ACL tear. The anterior tibial translation values were 3.34 ± 1.48 mm in injured knees and 0.86 ± 0.78 mm in uninjured knees. Side-to-side differences > 1 mm were found in 22 cases (95.65%). The diagnosis accuracy was 91.30% (95%CI: 71.96–98.92%) and sensitivity 95.45% (95%CI: 77.15–99.88%). The intraclass correlation coefficient showed an excellent test–retest reliability (ICC_3,1_ = 0.97 for the side-to-side difference in anterior tibial translation). The study highlights the accuracy and reliability of the dynamic ultrasound assessment of the anterior tibial translation in the diagnosis of unilateral anterior cruciate ligament tears. Ultrasound assessment is an accessible imaging tool that can provide valuable information and should be used together with physical examination in suspected cases of ACL injuries.

## 1. Introduction

Anterior cruciate ligament (ACL) tears are one of the most frequent ligament injuries of the knee, most of them needing surgical reconstruction [1,2]. In Romania, in 2015, there were 759 ACL/PCL reconstructions reported through the National ACL/PCL Reconstruction Register [3].

An accurate diagnosis of ACL injuries is essential for an appropriate treatment and a good prognosis. The American Academy of Orthopaedic Surgeons (AAOS) strongly recommends a detailed history and physical examination, as well as magnetic resonance imaging (MRI), for identifying ACL injuries [4]. However, the diagnostic accuracy of physical examination tests (anterior drawer test, Lachman test, pivot shift test) varies greatly in the literature [5]. Magnetic resonance imaging is considered highly accurate in diagnostic ACL tears [6]. However, performing MRI routinely for assessment of knee ligament injuries is not cost-effective and not always available [7]. Although arthroscopy is considered to be the gold standard for the diagnosis of ACL injuries, clinical diagnosis should be made with relevant imaging examinations [8]. In comparison with MRI, musculoskeletal ultrasound is more accessible, less expensive, with fewer impediments (e.g., metal implants, claustrophobia, pacemakers or other implants). Its reliability in assessing ligaments, tendons, muscles or joints has also been reported in several studies [9,10,11,12,13,14,15,16]. To date, there are several studies assessing the efficiency of ultrasound to identify ACL injuries. A systematic review performed by Wang et al. [17] showed that ultrasound can play a very important role in the diagnosis of ACL injury, although there are still some limitations, especially in identifying partial ACL tears (sensitivity of 15%).

In the literature, several ultrasound methods for ACL assessment are described that implies different patient positions (supine or prone, with the knee in different flexion degrees) and transducer placement (on the anterior or posterior aspect of the knee), static or dynamic evaluation, destabilizing strategies or different numbers of persons engaged in the examination [18]. One of the techniques used for the diagnosis of ACL injuries is the one described by Schwarz et al. [19], who used ultrasound to measure the anterior tibial translation to assess the ACL function. 

The aim of our study was to investigate the accuracy of dynamic ultrasound assessment of the anterior tibial translation, in diagnosing anterior cruciate ligament tears, and to assess its test–retest reliability. The accuracy of this method has been addressed in previous studies on acute ACL tears. We have evaluated the accuracy on injuries older than 4 weeks. To the best of our knowledge, the test–retest reliability of this assessment method used for the diagnosis of chronic ACL tears has not been studied.

## 2. Materials and Methods

### 2.1. Participants and Study Design

In this prospective study, all patients presenting to the clinic between January 2020–May 2021 with complaints of knee instability were screened for inclusion in the study. The inclusion criteria were: (1) age over 18 years; (2) positive Lachman test; (3) positive anterior drawer test; (4) history of knee trauma within the last 6 months. After selecting patients based on the above-mentioned inclusion criteria, only patients who underwent either MRI or arthroscopy were further included in the study (to confirm or to infirm the ACL tear). Exclusion criteria were: (1) acute knee injury (<4 weeks since the traumatic event); (2) positive posterior drawer test; (3) multidirectional instability; (4) previous knee surgery (including ACL reconstruction); (5) open knee wounds. Informed consent was obtained from all subjects who met the inclusion criteria and agreed to participate in the study. The study was carried out in accordance with the Declaration of Helsinki and was approved by the Institutional Ethics Committee (14b/28.02.2020).

### 2.2. Assessments

All patients underwent an initial clinical evaluation, followed by knee ultrasound assessment and MRI and arthroscopic ACL reconstruction if a total ACL injury was diagnosed.

**Figure 1:**
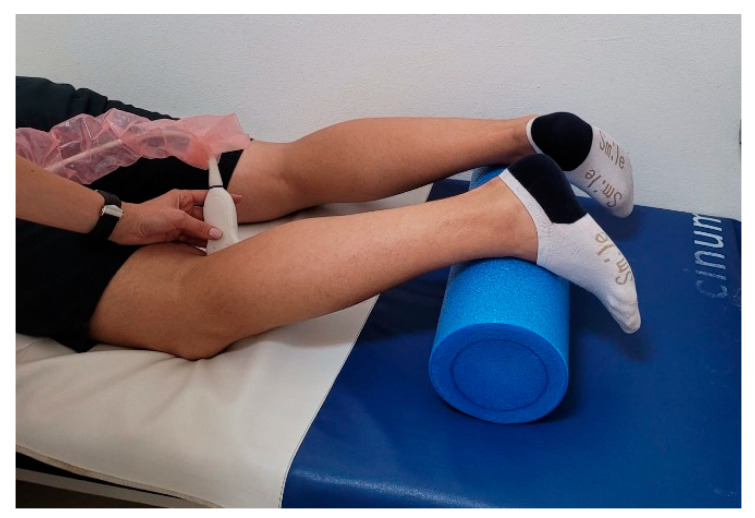
Patient position during ultrasound assessment.

**Figure 2:**
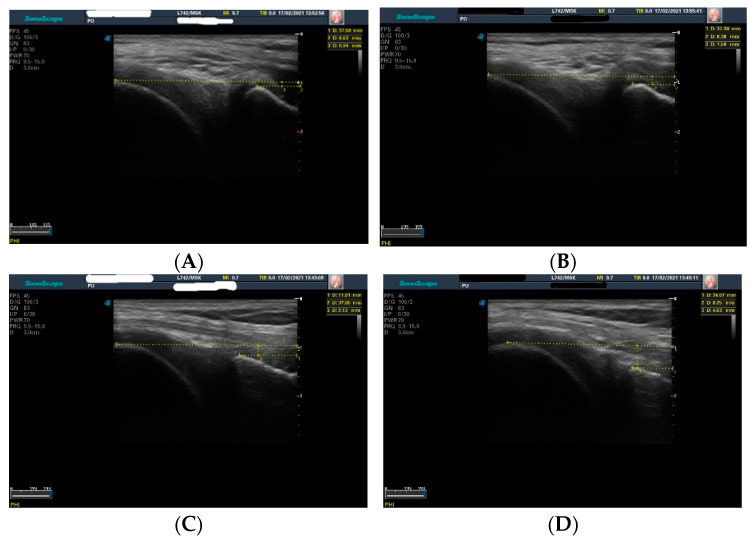
Anterior tibial translation measurements in uninjured and injured knee. (**A**) Static anterior tibial translation in uninjured knee (D1_uninjured_ = 0.94 mm); (**B**) Dynamic anterior tibial translation (manual pressure was applied in the proximal posterior aspect of the calf) in uninjured knee (D2_uninjured_ = 1.5 mm); (**C**) Static anterior tibial translation in injured knee (D1_injured_ = 2.13 mm); (**D**) Dynamic anterior tibial translation (manual pressure was applied in the proximal posterior aspect of the calf) in injured knee (D2_injured_ = 4.63 mm).

Knee ultrasound assessment was performed by an experienced orthopedic surgeon, using a Sonoscape S22 apparatus equipped with a 5–12 MHz linear-array transducer. The patient was lying prone with a roll under the lower legs in order to maintain the knee in 20° of flexion (Figure 1). Measurements were performed in both knees (injured and uninjured), at the postero-medial aspect of the knee. The distance between the tangent line to the medial femoral condyle and the tangent to the posterior aspect of the tibia was measured in static position (D1) and after applying manual pressure on the posterior proximal aspect of the calf (D2) [20] (Figure 2). For each knee, the difference between the two distances was calculated (D2 − D1). The translation differences between the injured and uninjured knee were calculated: ΔD = (D2_injured_ – D1_injured_) – (D2_uninjured_ – D1_uninjured_) [20]. Schwarz et al. reported that a value greater than 1 mm for ΔD is a reliable threshold for the diagnosis of an ACL tear [19,20]. The measurements were repeated after 15 min, in order to assess the test–retest reliability.

### 2.3. Statistical Analysis

Statistical analysis was performed with MedCalc Statistical Software version 20.014 (MedCalc Software Ltd., Ostend, Belgium). Data were tested for normality using Shapiro–Wilk test and descriptive statistics were performed. In order to assess the sensitivity and accuracy of the ultrasound-based diagnostic for complete ACL tear, a 2 × 2 contingency table was created, comparing the results from ultrasound with those obtained by arthroscopy. Intraclass correlation coefficient (ICC_3,1_) was used to assess the test–retest reliability [21]. ICC values greater than 0.90 were considered as excellent, values between 0.75 and 0.90 as good and values less than 0.75 as moderate [21]. Standard error of measurement (SEM) was calculated according to the formula $\mathrm{SEM}=\mathrm{SDpooled}\;\times \;\sqrt{1-\mathrm{ICC}}$ [22,23]. The measurements are considered more reliable if the SEM values are smaller [22]. The smallest detectable change at 95% confidence interval (SDC_95_) assessed the magnitude of the real change between measurements necessary to exceed error. The lower the values for SDC_95_ are, the higher the reliability is [24]. SDC_95_ is calculated using the formula: ${\mathrm{SDC}}_{95}=1.96\times \sqrt{2}\times \mathrm{SEM}$ [23]. A paired sample *t*-test was performed in order to assess the systematic bias [22,23]. Statistical significance was set *p* < 0.05 for all tests.

## 3. Results

Twenty-three patients who met the inclusion criteria, agreed to participate in the study and were also evaluated by MRI and arthroscopy were included in the study. Mean age was 32 ± 8.42 years; 16 patients were males (69.56%). The traumatic event took place during amateur sporting activities (soccer—9 cases (39.13%); ski—10 cases (43.48%); basketball—2 cases (8.7%)) or professional sport activities (handball—1 case (4.34%); soccer—1 case (4.34%)).

The anterior tibial translations measured by ultrasound for the injured and non-injured knee, in both measurement sessions, are present in Table 1.

**Figure 3:**
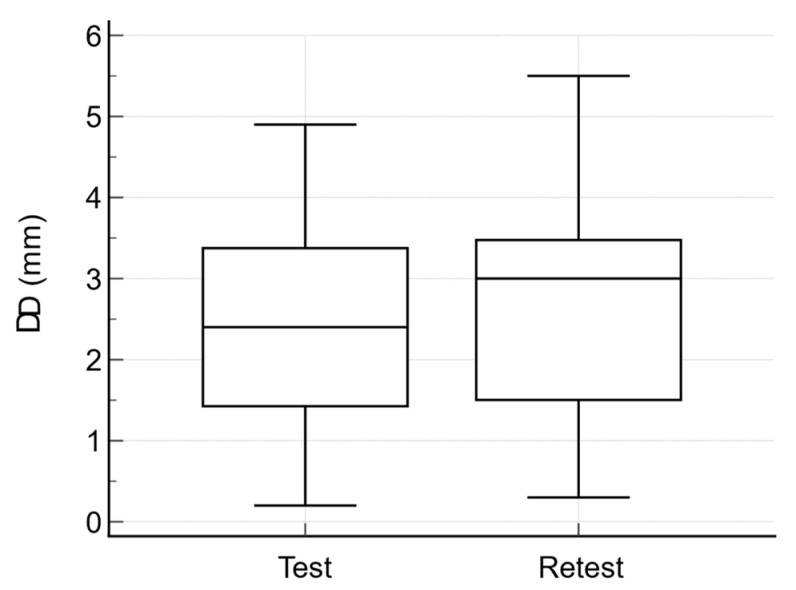
Side-to-side differences in tibial translation (ΔD).

The side-to-side difference in tibial translation (ΔD) was greater than 1 mm in 22 cases (95.65%), with a mean of 2.47 ± 1.25 mm at the first measurement, and 2.71 ± 1.39 mm at retest (Figure 3). Complete ACL tears were confirmed in all 23 cases by MRI, and in 22 cases by arthroscopy (including the case non-confirmed by ultrasound). One case was diagnosed as partial ACL tear by arthroscopy and no ACL reconstruction was performed. Using the threshold of 1 mm for side-to-side differences of tibial translation, complete ACL tears have been correctly diagnosed in 22 cases, the sensitivity of the method being 95.45% (95%CI: 77.15–99.88%) and the accuracy 91.30% (95%CI: 71.96–98.92%).

Significant differences were found between test and retest values for the anterior tibial translation, for both injured (*p* = 0.0002) and uninjured knees (*p* = 0.01).

The ICC_3,1_, SEM and SDC_95_ values for D1 and D2 measurement, for both injured and uninjured knee, as well as for ΔD, are presented in Table 2. Lower values for SEM and SDC_95_ were observed for both measurements (D1 and D2) in the uninjured knee.

## 4. Discussion

Although ultrasound is a frequently used method in the diagnosis of musculoskeletal injuries, in both orthopedics and rehabilitation medicine [25,26], ultrasound assessments for the diagnosis of ACL injuries showed varied results, mainly due to the different methodologies used (conventional ultrasound and functional/dynamic ultrasound) [7]. The aim of the present study was to investigate the accuracy of dynamic ultrasound assessment of the anterior tibial translation, in diagnosing anterior cruciate ligament tears, and to assess its test–retest reliability. We found a diagnostic accuracy of dynamic ultrasound measurement of anterior tibial translation of 91.30% and a sensitivity of 95.45% for the diagnosis of complete ACL tears.

The anterior tibial translation measurements showed a systematic bias between test–retest, for both injured and uninjured knees. Since these measurements were only taken 15 min apart, this is not surprising, given the viscoelastic properties of knee ligaments [27]. These findings suggest that the ligaments in the knee become stretched during the anterior tibial translation measurement, and the ligaments do not return to their original length within 15 min (the amount of time between test and retest). These findings also suggest that the manual pressure on the posterior proximal aspect of the calf applied by the tester is likely causing this systematic lengthening of the knee ligaments. Such pressure should be controlled to minimize variations among systematic lengthening of these ligaments during the test. Future studies should examine the reliability of this anterior tibial translation measurement with 2–3 days between the test and retest, rather than 15 min, to understand the full scope of reliability, systematic variability, and sensitivity of this assessment. Furthermore, future studies should also examine the time course of ligament lengthening to understand how long it takes for the affected ligaments to return to their original length between successive measurements. Such information would improve recommendations on how much time to wait between anterior tibial translation measurements when multiple tests are required.

In our study, we used the same method as Palm et al. [20], and found similar results in terms of sensitivity. In their study, Palm et al. [20] investigated if an examiner with basic expertise in ultrasonography and little or no experience in arthrosonography can use functional ultrasonography with the same high diagnostic accuracy as an experienced sonographer. They found a sensitivity of 97% for the method, the ACL tears being confirmed by arthroscopy in 32 of 33 cases.

Kumar et al. [28] also found a high sensitivity for this method (81.65%), after the assessment of 130 patients with a non-acute knee injury. They compared the efficacy of dynamic ultrasound in identifying ACL tears with MRI.

Gebhard et al. [29] used the ultrasound to quantify the anterior tibial translation in their study, the difference between the two methods being the way the tibial translation was induced. We used the same method as Palm et al. [20] in their study, applying manual pressure on the posterior proximal aspect of the calf. Gebhard et al. [29] manually lifted the lower leg under ultrasound control as far as possible, while the thigh remained in contact with the surface; after marking the tibial head, the lower leg was carefully released and drawn by gravity into anterior draw position. They opted for the gravity-induced anterior tibial translation as they noticed pain during dorsal force on most of the acute ACL lesions. For a minimum intra-individual difference of 5 mm, the authors reported a sensitivity of 96% [29].

We found excellent test–retest reliability in the dynamic ultrasound assessment of the anterior tibial translation, with patients lying prone and measurements being made in the postero-medial aspect of the knees (ICC_3,1_ = 0.97 for the side-to-side difference in anterior tibial translation). A good intra-rater reliability of ultrasound assessment of the anterior tibial translation was reported by Teng et al. [30], but in participants with no ACL tears.

For the diagnosis of ACL injuries, direct and indirect signs, as well as signs of antero-posterior instability under ultrasound imaging were proposed, with different diagnosis accuracy and sensitivity [31]. As in physical examination, the anterior tibial translation is used as a sign of antero-posterior instability under ultrasound assessment, with greater values in the presence of ACL injuries compared to uninvolved knees [19,20,28,29]. In our study the anterior tibial translation of the affected knees was also greater than in uninjured knees (3.34 ± 1.48 mm vs. 0.86 ± 0.78 mm), similar to values found by Palm et al. [20] (3.8 ± 1.5 mm vs. 0.1 ± 0.7 mm). The values reported by Kumar et al. [28] for the anterior tibial translation for the injured and uninjured knee were slightly greater than our results (4.21 ± 2.93 mm and 2.16 ± 2.67 mm, respectively). Gebhard et al. [29] reported a mean anterior displacement of the medial tibial plateau of 14.1 ± 3.5 mm in ACL-injured knees and of 8.3 ± 3.4 mm in uninjured knees, values greater than those reported by us, Palm et al. [20] and Kumar et al. [28]. One explanation could be the different method of inducing anterior tibial translation. Using a different method (patient in a seated position, with the knee flexed to about 70–80°, the transducer placed onto the anterior aspect of the knee, above the level of tibial tuberosity, parallel with the patellar tendon; examiner in front of the patient; the patient’s lower leg was pushed backwards with the examiner’s foot), Grzelak et al. [32] reported absolute knee anterior translation values of 8.67 ± 2.65 mm in injured knees and 2.88 ± 1.26 mm in uninjured knees.

Schwarz et al. reported that a value greater than 1 mm for side-to-side differences in ultrasound-measured anterior tibial translation is a reliable threshold for the diagnosis of an ACL tear [19,20]. We found a side-to-side difference in tibial translation (ΔD) greater than 1 mm in 22 cases (95.65%), with a mean of 2.47 ± 1.25 mm. Palm et al. [20] and Kumar et al. [28] have also considered the threshold of 1 mm in side-to-side difference for anterior tibial translation for a diagnosis of ACL tear.

The examiner in our study was an orthopedic surgeon with significant prior experience with the use of ultrasound. However, the results from Palm et al. [20] showed that an examiner without specialist knowledge in ultrasonography can accurately diagnose acute ACL injuries using functional ultrasonography.

Ultrasound assessment of anterior tibial translation, as a side-to-side difference of more than 1 mm, proved to be accurate and reliable for the diagnosis of complete ACL tears. To the best of our knowledge, this is the first study that has assessed the test–retest reliability of the dynamic ultrasound assessment of anterior tibial translation for the diagnosis of chronic ACL tears. Our results showed an excellent test–retest reliability for this method. However, the method can be used only if the injury is unilateral, and this is a limitation. Further studies are needed to find a threshold value for the anterior tibial translation, to use it also in cases in which there is a laxity or instability in the contralateral knee and also to test the inter-rater reliability of this method. Some limitations of this study have to be considered. We have not evaluated acute ACL tears. Not examining the side-to-side differences with another device, such as an arthrometer, could also be a limitation of the study.

## 5. Conclusions

The study highlights the accuracy and reliability of the dynamic ultrasound assessment of the anterior tibial translation, in the diagnosis of unilateral anterior cruciate ligament tears. Ultrasound assessment is an accessible imaging tool that can provide valuable information and should be used together with the physical examination in suspected cases of ACL injuries.

## Figures and Tables

**Table 1:** Anterior tibial translation in injured and non-injured knee.

	Injured Knee	*p* *	Uninjured Knee	*p* **
Anterior tibial translation (D2 − D1), mm (mean ± SD)	3.34 ± 1.48	0.0002	0.86 ± 0.78	0.01
Anterior tibial translation (D2 − D1) − retest, mm (mean ± SD)	3.66 ± 1.64		0.95 ± 0.78	

*p* *—relates to the differences between test and retest for the injured knee; *p* **—relates to the differences between test and retest for the uninjured knee.

**Table 2:** The ICC_3,1_, SEM and SDC_95_ values for D1, D2 measurements and ΔD.

	ICC_3,1_	95%CI	SEM	SDC_95_
D1 injured knee	0.99	0.997–0.999	0.23	0.63
D2 injured knee	0.99	0.98–0.99	0.31	0.85
D1 uninjured knee	0.99	0.991–0.998	0.08	0.22
D2 uninjured knee	0.98	0.973–0.995	0.18	0.49
ΔD	0.97	0.945–0.99	0.22	0.6

ICC_3,1_—intraclass correlation coefficient; 95%CI—95% confidence interval; SEM—standard error of measurement; SDC_95_—smallest detectable change at 95% confidence interval; SEM and SDC_95_ are expressed in the same measurement units as the test.
